# Alternative source of stem cells derived from human periodontal ligament: a new treatment for experimental autoimmune encephalomyelitis

**DOI:** 10.1186/s13287-015-0253-4

**Published:** 2016-01-04

**Authors:** Oriana Trubiani, Sabrina Giacoppo, Patrizia Ballerini, Francesca Diomede, Adriano Piattelli, Placido Bramanti, Emanuela Mazzon

**Affiliations:** Stem Cells and Regenerative Medicine Laboratory, Department of Medical, Oral and Biotechnological Sciences, University “G. d’Annunzio”, Chieti-Pescara, via dei Vestini, 31, 66100 Chieti, Italy; IRCCS Centro Neurolesi “Bonino-Pulejo”, Via Provinciale Palermo, contrada Casazza, 98124 Messina, Italy; Department of Psychological, Humanities and Territorial Sciences, University “G. d’Annunzio” Chieti-Pescara, Chieti, Italy

**Keywords:** Stem cells derived from human periodontal ligament, Multiple sclerosis, Neurotrophic factors, Apoptosis

## Abstract

**Background:**

Multiple sclerosis is a demyelinating disease mostly of autoimmune origin that affects and damages the central nervous system, leading to a disabling condition. The aim of the present study was to investigate whether administration of mesenchymal stem cells from human periodontal ligament (hPDLSCs) could ameliorate multiple sclerosis progression by exerting neuroprotective effects in an experimental model of autoimmune encephalomyelitis (EAE).

**Methods:**

EAE was induced by immunization with myelin oligodendroglial glycoprotein peptide (MOG)_35–55_ in C57BL/6 mice. After immunization, mice were observed every 48 hours for signs of EAE and weight loss. At the onset of disease, approximately 14 days after immunization, EAE mice were subjected to a single intravenous injection of hPDLSCs (10^6^ cells/150 μl) into the tail vein. At the point of animal sacrifice on day 56 after EAE induction, spinal cord and brain tissues were collected in order to perform histological evaluation, immunohistochemistry and western blotting analysis.

**Results:**

Achieved results reveal that treatment with hPDLSCs may exert neuroprotective effects against EAE, diminishing both clinical signs and histological score typical of the disease (lymphocytic infiltration and demyelination) probably through the production of neurotrophic factors (results focused on brain-derived neurotrophic factor and nerve growth factor expression). Furthermore, administration of hPDLSCs modulates expression of inflammatory key markers (tumor necrosis factor-α, interleukin (IL)-1β, IL-10, glial fibrillary acidic protein, Nrf2 and Foxp3), the release of CD4 and CD8α T cells, and the triggering of apoptotic death pathway (data shown for cleaved caspase 3, p53 and p21).

**Conclusions:**

In light of the achieved results, transplantation of hPDLSCs may represent a putative novel and helpful tool for multiple sclerosis treatment. These cells could have considerable implication for future therapies for multiple sclerosis and this study may represent the starting point for further investigations.

## Background

Multiple sclerosis (MS) is a chronic inflammatory and demyelinating disease of the central nervous system (CNS). In northern industrialized countries, MS affects about 0.1 % of the population and is the first cause of disability of non-traumatic origin in young adults [1]. The etiology of MS is still incompletely understood; however, it seems that the main etiopathogenic event is represented by an unusual response of the immune system cells (T and B lymphocytes) against myelin sheaths of neurons [2].

To date, current treatments for MS only offer palliative relief without providing a cure, and many are also associated with adverse effects that limit their long-term utility [3].

Recently, the potential role of mesenchymal stem cells (MSCs), derived especially from bone marrow, in promoting tissue repair and disease control has been investigated by using an experimental autoimmune encephalomyelitis (EAE) model [4–7], the most common animal model that mimics the main features of human MS [8].

It is well known that MSCs can differentiate under certain circumstances, giving rise to various neuronal and glial cell lineages [9]. Also, MSC transplantation is able to modulate the immune system at CNS lesions and enhances remyelination and the repairing process [10, 11]. However, the defined immunomodulatory mechanisms by which these cells exert their regulatory activities have not been clarified yet. It is probable that neuroprotective effects may result from release of anti-apoptotic proteins as well as neurotrophic factors [12, 13].

Although the therapeutic efficacy of these cells has been widely proven, we looked to an alternative source of stem cells that could be less invasive for removal. In this regard the mesenchymal stem cells derived from the oral cavity and in particular from human periodontal ligament (hPDLSCs) may represent a very promising source.

The hPDLSCs are isolated from the periodontium, a tissue of ectomesenchymal origin that is particularly well adapted to different functions; it supports the teeth in their sockets and at the same time permits withstanding the considerable forces of mastication. In particular, the periodontium tissue contains cell islands called epithelial rests of Malassez regulating the growth induction of nerve endings [14].

The hPDLSCs originate from cranial neural crest cells, a transient embryonic structure, composed of migratory and multipotent stem cells that provide a wide variety of cell phenotypes including peripheral neurons, Schwann cells, and vascular smooth muscle cells. They represent an available niche of MSCs that can be obtained with restricted morbidity and without additional risks to the donor using minimally invasive periodontal access flap surgery [15–18]. The hPDLSCs can differentiate under appropriate stimuli into mesenchymal tissues such as bone, cartilage, fat and into neurogenic lineage [18–21].

With regard to the neuronal differentiation, many studies have reported on the successes of in vitro formation of neuronal lineage cells from PDLSCs [20, 22] and in particular it has also been shown that untreated oral stem cells were able to differentiate into functional neurons upon injection into the developing avian nervous system [23].

Furthermore, the hPDLSCs express proteins implicated in the cell cycle regulation and stress response, homing, detoxification, neurogenesis and neuronal function homeostasis suited for their neural crest origin [24]. Moreover, in addition to their self-renewal potential and multilineage differentiation ability, dental MSCs possess potent immunomodulatory functions and a higher capability of cell growth in comparison to bone marrow-derived MSCs, representing a new stem cell niche for translational medicine [25, 26].

This study aimed to investigate the possible neuroprotective effect of hPDLSC administration against the overall cascade of events occurring after EAE induction in C57BL/6 mice by improving development and clinical signs of disease.

## Methods

### Ethics statement

The protocol and informed consent from human periodontal ligament biopsies were approved by the Medical Ethics Committee at the Medical School, “G. d’Annunzio” University, Chieti, Italy (n°266/17.04.14). The formal consent form was signed by the subjects (range age 20–35 years) before specimen collection. The Department of Medical, Oral and Biotechnological Sciences and the Laboratory of Stem Cells and Regenerative Medicine are certified according to the quality standard ISO 9001:2008 (certificate n° 32031/15/S).

### Isolation and culture of hPDLSCs

Five human periodontal ligament biopsies were scraped from human premolar teeth that were due to be removed for orthodontic treatment in healthy patients. The periodontal ligament tissue was obtained by grating the roots using the Gracey’s curette [18]. The samples were washed five times with phosphate-buffered saline (PBS; LiStarFish), and cultured using TheraPEAK™MSCGM-CD™ BulletKit serum free, which was chemically defined (MSCGM-CD) medium for the growth of human MSCs (Lonza, Basel, Switzerland). The medium was changed twice a week, and cells migrating from the explant tissue after reaching about 80 % confluence were trypsinized (LiStar Fish) and subcultured until passage 2.

### hPDLSC immunophenotyping

hPDLSCs at the second passage were collected; 5 × 10^5^ cells per sample were incubated with 1 μg of the specific antibody, conjugated with fluorescein isothiocyanate, phycoerythrin, allophycocyanin, phycoerythrin-cyanine 5.5, or Alexa Fluor 488 for 30 min at 4 °C in the dark. hPDLSCs were stained using the following antibodies: anti-CD13, anti-CD29, anti-CD44, anti-CD45, anti-CD105, anti-CD166 (Ancell, MN, USA), anti-CD14, anti-CD133 (BergischGladbach, Germany), anti-CD73, anti-CD90, anti-CD117, anti-CD146, anti-CD271, anti-Sox2, anti-HLA-DR, anti-SSEA4, anti-OCT3/4 (Becton Dickinson, BD, San Jose, CA, USA), anti-CD144 (Acris Antibodies, Herford, Germany) and anti-CD34 (Beckman Coulter, Fullerton, CA, USA). After incubation, cells were acquired with a flow cytometer (FACS Calibur; BD). Data were analyzed by the FlowJo software v8.8.6 (TreeStar, Ashland, OR, USA) [17].

### Morphological analysis

Glass-adherent hPDLSCs at passage 2 were fixed with 2.5 % glutaraldehyde in 0.1 M cacodylate buffer, pH7.4, for 2 h, and were subsequently stained with toluidine blue and observed by light microscopy. All sections were observed with a Zeiss Axiophot apparatus, and images captured using a Nikon digital camera Digital Sight.

### MTT assay

The viability of hPDLSC culture was analyzed by the quantitative colorimetric MTT (3-[4,5-dimethyl-2-thiazolyl]-2,5-diphenyl-2Htetrazoliumbromide test) (Promega, Milan, Italy). Cells (2 × 10^3^ cells/well) were seeded into a 96-well culture plate with MSCGM-CD, and the supernatants were read at 650 nm wavelength using a ND-1000 NanoDrop Spectrophotometer (NanoDrop Technologies, Rockland, DE, USA). The MTT assay was performed in five independent experiments, one for each donor, with five replicates for each experimental point.

### Mesengenic differentiation and histochemical analysis

For osteogenic and adipogenic differentiation, hPDLSCs at passage 2 were incubated in MSCGM-CD (Lonza) medium with osteogenic supplements and in adipogenesis induction/maintenance medium (Lonza), respectively. Osteogenic and adipogenic induction was confirmed by means of colorimetric assay as previously described by Trubiani et al. [17]. hPDLSCs for chondrogenic differentiation at passage 2 were transferred into 15 mL polypropylene tubes and centrifuged at 800 rpm for 8 min to obtained pellets at the bottom of the tube. A pellet was incubated in hMSC Chondro Bullet Kit (Lonza) with added transforming growth factor β3, as described by Yang et al. [27]. The pellets were fixed in 4 % paraformaldehyde at 4 °C for 24 h, dehydrated in an ascending series of ethanols (40, 70, 90 and 100 % ethanol; 20 min/step) and embedded in paraffin. Sections (3 μm) were cut and stained with 1 % Alcian blue (pH2.5; Sigma Aldrich, Milan, Italy) for 5 min and observed by means of light microscopy. The differentiation process was performed in five separate experiments using hPDLSCs derived from five different donors.

### RNA isolation and real time-PCR analysis

Osteogenic and adipogenic markers were evaluated by real-time polymerase chain reaction (PCR). To this end, total RNA was isolated using the Total RNA Purification Kit (NorgenBiotek Corp., Ontario, CA, USA) according to the manufacturer’s instructions. The M-MLV Reverse Transcriptase reagents (Applied Biosystems) were used to generate cDNA. Real-Time PCR was carried out with the Mastercycler ep realplex real-time PCR system (Eppendorf, Hamburg, Germany). hPDLSC expression of Runt-related transcription factor-2 (RUNX-2) and alkaline phosphatase (ALP) was evaluated after 7 days in osteogenic differentiated culture. The expression of fatty acid binding protein 4 (FABP4) and peroxisome proliferator-activated receptor γ (PPARγ) were analyzed after 28 days of adipogenic differentiation culture. Aggrecan (ACAN) and collagen type II (COL2A1) expression were evaluated after 28 days of chondrogenic induction. Commercially available TaqMan Gene Expression Assays (RUNX-2 Hs00231692_m1; ALP Hs01029144_m1; FABP4 Hs01086177_m1; PPARγ Hs01115513_m1; ACAN Hs00153936_m1; COL2A1 Hs00264051_m1) and the TaqMan Universal PCR Master Mix (Applied Biosystems, Foster City, CA, USA) were used according to standard protocols. Beta-2 microglobulin (B2M Hs99999907_m1) (Applied Biosystems, Foster City, CA, USA) was used for template normalization. Real-time PCR was performed in three independent experiments, and duplicate determinations were carried out for each sample.

### Animals

Male C57BL/6 mice (Harlan, Milan, Italy), 12 weeks of age and weighing 20–25 g, were housed in individually ventilated cages with food and water ad libitum. The room was maintained at a constant temperature and humidity with a 12 h/12 h light/dark cycle.

### Ethics statement

This study was carried out in strict accordance with the recommendations in the guide for the care and use of laboratory animals of the National Institutes of Health. The protocol was approved by the Ministry of Health “General Direction of animal health and veterinary drug” (Authorization 621/2015). In particular, animal care was in compliance with Italian regulations on the protection of animals used for experimental and other scientific purposes (D.lgs 26/2014).

### Induction of EAE

After anesthesia, induced with an anesthetic cocktail composed of tiletamine plus xylazine (10 ml/kg, intraperitoneally), EAE was actively induced using myelin oligodendrocyte glycoprotein peptide (MOG)_35–55_ (MEVGWYRSPFSRVVHLYRNGK; % peak area by HPLC ≥95, AnaSpec, EGT Corporate Headquarters, Fremont, CA, USA), according to Paschalidis et al. [28]. Mice were immunized subcutaneously with 300 μl/flank of the emulsion consisting of 300 μg MOG_35–55_ in PBS mixed with an equal volume of complete Freund’s adjuvant containing 300 μg heat-killed *M. Tubercolosis* H37Ra (Difco Laboratories Sparks, MD, USA). Immediately after MOG_35–55_ injection, the animals received an intraperitoneal injection of 100 μl *B. Pertussis* toxin (Sigma-Aldrich; 500 ng/100 μl), repeated 48 h later. The disease follows a course of progressive degeneration, with visible signs of pathology consisting of flaccidity of the tail and loss of motion of the hind legs.

### Experimental design

Mice were randomly allocated into the following groups (n = 30 total animals):

Naive group (n = 10)—mice did not receive MOG_35–55_ or other treatment;

EAE group (n = 10)—mice subjected to EAE as described above;

EAE + hPDLSC group (n = 10)—at the onset of disease signs that normally occurs approximately 14 days after immunization with MOG_35–55_, EAE mice were subjected to a single intravenous injection into the tail vein with hPDLSCs (10^6^ cells/150 μl).

hPDLSCs from the five donor lines were randomly assigned to each animal given that they showed similar phenotypic and morphological features as well as growth and multidifferentiation ability.

Animals were observed every 48 h for signs of EAE and weight loss. At the end of the experiment, which occurred at day 56 after EAE induction, all animals treated with hPDLSCs were euthanized with intraperitoneal Tanax (5 ml/kg body weight).

Furthermore, spinal cord and brain tissues were sampled and processed in order to evaluate parameters of the disease.

### Clinical disease score and body weight evaluation

The first measurement of clinical disease score was taken on the day of EAE induction (day 0), and all the subsequent measurements were recorded every 48 h until sacrifice. Clinical score was evaluated using a standardized scoring system [29] as follows: 0 = no signs; 1 = partial flaccid tail; 2 = complete flaccid tail; 3 = hind limb hypotonia; 4 = partial hind limb paralysis; 5 = complete hind limb paralysis; 6 = moribund or dead animal. Animals with a score ≥5 were sacrificed to avoid animal suffering.

In addition, the first measurement of body weight was taken on the day of EAE induction (day 0), and all the subsequent measurements were recorded every 48 h until sacrifice. The variation in body weight has been expressed compared to the day of EAE induction (day 0); also the value has been expressed as mean ± SEM of all animals for each experimental group.

### Luxol Fast Blue

To show myelin and phospholipids in histological sections, Luxol Fast Blue (LFB) staining was performed according to the manufacturer’s protocol (Bio-Optica, Milan, Italy). The staining provides myelin in turquoise blue, neurons and glial nuclei in pink/violet and Nissl substance in pale pink.

### Light microscopy

At 56 days after EAE induction, spinal cords were sampled from the cervical region to the lumbar region, fixed in 10 % (w/v) in PBS-buffered formaldehyde, embedded in paraffin and then cut into 7 μm sections. The sections were deparaffinized with xylene, rehydrated, and stained with hematoxylin and eosin (H&E) to be studied by optical microscope (Leica microscope ICC50HD).

### Immunohistochemical evaluation

After deparaffinization with xylene, sections of spinal cord samples were hydrated. Detection of glial fibrillary acidic protein (GFAP), interleukin (IL)-1β, IL-10, CD4 and CD8α was carried out after boiling in citrate buffer 0.01 M pH 6 for 4 min. Endogenous peroxidase was quenched with 0.3 % (v/v) hydrogen peroxide in 60 % (v/v) methanol for 30 min. Nonspecific adsorption was minimized by incubating the section in 2 % (v/v) normal goat serum in PBS for 20 min.

Sections were incubated overnight with:

- anti-GFAP monoclonal antibody (1:50 in PBS v/v; Cell Signaling Technology);
- anti-IL-1β polyclonal antibody (1:100 in PBS v/v; Santa Cruz Biotechnology, Inc);
- anti-IL-10 (1:100 in PBS v/v; Santa Cruz Biotechnology, Inc);
- anti-CD4 polyclonal antibody (1:50 in PBS v/v; Santa Cruz Biotechnology, Inc);
- anti-CD8α polyclonal antibody (1:50 in PBS v/v; Santa Cruz Biotechnology, Inc).

Endogenous biotin or avidin binding sites were blocked by sequential incubation for 15 min with biotin and avidin (DBA, Milan, Italy), respectively. Sections were washed with PBS and incubated with secondary antibody. Specific labeling was detected with a biotin-conjugated goat anti-rabbit IgG and avidin–biotin peroxidase complex (Vectastain ABC kit, VECTOR). The immunostaining was developed with peroxidase substrate kit DAB (Vector Laboratories, Inc.) (brown color) and counterstaining with hematoxylin (blue background).

To verify the binding specificity, some sections were also incubated with only the primary antibody (no secondary) or with only the secondary antibody (no primary). In these cases, no positive staining was found in the sections, indicating that the immunoreaction was positive in all the experiments carried out.

All sections were obtained using light microscopy (LEICA DM 2000 combined with LEICA ICC50 HD camera). Leica Application Suite V4.2.0 software was used as the image computer program to acquire immunohistochemical pictures.

### Western blot analysis

All the extraction procedures were performed on ice using ice-cold reagents. In brief, spinal cord and brain tissues were suspended in extraction buffer containing 0.32 M sucrose, 10 mM Tris-HCl, pH 7.4, 1 mM EGTA, 2 mM EDTA, 5 mM NaN_3_, 10 mM 2-mercaptoethanol, 50 mM NaF, and protease inhibitor tablets (Roche Applied Science, Monza, Italy), and they were homogenized at the highest setting for 2 min. The homogenates were chilled on ice for 15 min and then centrifuged at 1000 g for 10 min at 4 °C, and the supernatant was collected to evaluate the content of cytoplasmatic proteins.

The pellets were suspended in the supplied complete lysis buffer containing 1 % Triton X-100, 150 mM NaCl, 10 mM Tris-HCl, pH 7.4, 1 mM EGTA, 1 mM EDTA protease inhibitors (Roche), and then were centrifuged for 30 min at 15,000 g at 4 °C. Then, supernatant containing nuclear extract was collected to evaluate the content of nuclear proteins. Supernatants were stored at –80 °C until use. Protein concentration in homogenate was estimated by Bio-Rad Protein Assay (Bio-Rad, Segrate, Italy) using BSA as standard, and 30 μg of cytosol and nuclear extract from each sample were analyzed.

Proteins were separated on sodium dodecyl sulfate-polyacrylamide minigels and transferred onto PVDF membranes (Immobilon-P Transfer membrane, Millipore), blocked with PBS containing 5 % nonfat dried milk (PM) for 45 min at room temperature, and subsequently probed at 4 °C overnight with specific antibodies for tumor necrosis factor (TNF)-α (1:500; Cell Signaling Technology), Nrf2 (Santa Cruz Biotechnology, Inc), FOXP3 (1:50; Cruz Biotechnology, Inc), cleaved-caspase 3 (1:500; Cell Signaling Technology), p21 (1:1000; Millipore), p53 (1:1000; Abcam), brain-derived neurotrophic factor (BDNF; 1:200; Santa Cruz Biotechnology, Inc), and nerve growth factor (NGF; 1:250; Abcam) in 1× PBS, 5 % (w/v) nonfat dried milk, 0.1 % Tween-20 (PMT). HRP-conjugated goat anti-mouse IgG or HRP-conjugated goat anti-rabbit IgG were incubated as secondary antibody (1:2000; Santa Cruz Biotechnology Inc) for 1 h at room temperature. To ascertain that blots were loaded with equal amounts of protein lysates, they were also incubated with antibody for GAPDH HRP Conjugated (1:1000; Cell Signaling Technology). The relative expression of protein bands was visualized using an enhanced chemiluminescence system (Luminata Western HRP Substrates, Millipore) and protein bands were acquired and quantified with ChemiDoc™ MP System (Bio-Rad) and a computer program (ImageJ software), respectively.

Blots are representative of three separate and reproducible experiments. The statistical analysis was carried out on three repeated blots performed on separate experiments.

### Statistical evaluation

GraphPad Prism version 6.0 program (GraphPad Software, La Jolla, CA, USA) was used for statistical analysis of the data.

The factors under investigation were the time elapsed and the mRNA expression for MTT assay and the mesengenic differentiation, respectively. Data are expressed as means and standard deviation of the recorded dependent variables: the optical density (MTT assay) and mRNA expression (osteogenic, adipogenic and chondrogenic differentiation). The differences among the levels of the two factors under investigation were evaluated performing four distinct two-way analysis of variance tests, one for each experiment. Tukey tests were applied for pairwise comparisons. A value of *p* < 0.05 was considered statistically significant in all tests.

The results for clinical disease score, body weight and western blot analysis were statistically analyzed using one-way analysis of variance followed by a Bonferroni post hoc test for multiple comparisons. A *p* value less than or equal to 0.05 was considered significant. Results are expressed as the mean ± SEM of n experiments.

## Results

### Morphological investigations of hPDLSCs

**Fig. 1:**
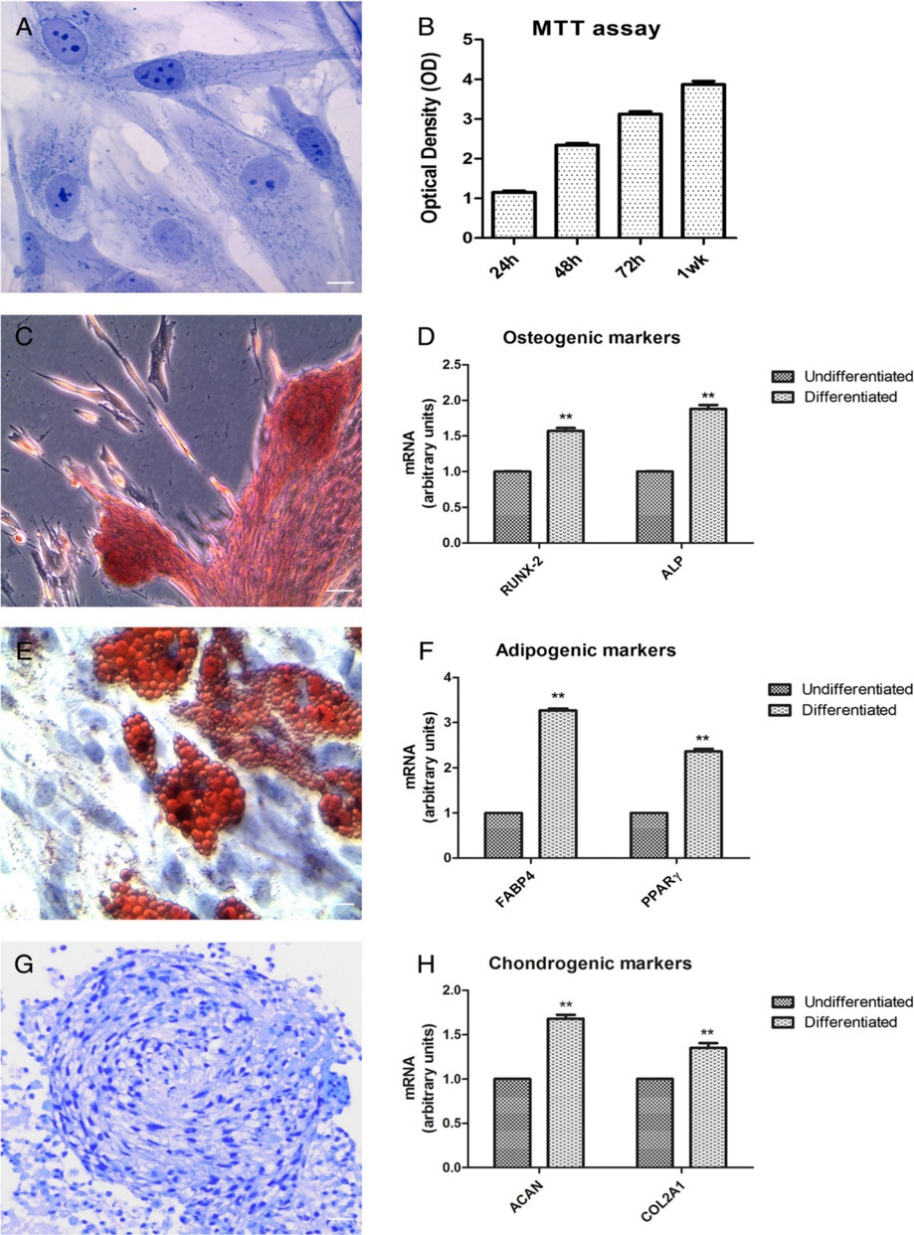
**a** Toluidine blue staining of primary cultures of hPDLSCs observed by light microscopy. The cells show mainly a spindle-shaped appearance with long cytoplasmatic processes, euchromatic nuclei with one or more nucleoli and rough endoplasmic reticulum profiles. Original magnification: 40×. **b** Proliferation rate is assessed by MTT assay in five hPDLSCs grown with xeno-free medium. The results are expressed as mean ± SEM of three independent experiments, and five replicates for each experimental point. The proliferation rate is measured as the absorbance detected at 650 nm OD. **c** The hPDLSCs, induced to osteogenic differentiation and stained with Alizarin Red S, show a characteristic arrangement; after 4 weeks the cells break off from the bottom plate and several areas of high levels of mineralization referred to as bone nodules are evident. Original magnification: 10×. **d** The bar graph shows mRNA levels, determined by real-time PCR, of osteo-related genes, i.e., alkaline phosphatase (*ALP*) and Runt-related transcription factor-2 (*RUNX2*) at 7 days of culture. **e** Adipogenic commitment has been evaluated by the appearance of oil-red O-positive lipid vacuoles. Original magnification: 40×. **f** The adipo-related genes, i.e., fatty acid binding protein 4 (*FABP4*) and peroxisome proliferator-activated receptor γ (*PPARγ*), analyzed by real-time PCR are shown. **g** Alcian blue staining of the chondrogenic pellets obtained from hPDLSCs indicating the chondrogenic differentiation. **h** Effect of chondro-inductive medium on the expression of chondrogenic differentiation-related genes, such as aggrecan (*ACAN*) and collagen type II (*COL2A1*), was analyzed by real-time PCR. **p < 0.05. Scale bars = 10 μm. For each experiment a representative image has been shown

Primary cultures of hPDLSCs at second passage, observed by light microscopy, were stained with Toluidine blue. Adherent cells to glass cover slips showed a spindle-shaped morphology with elongated cytoplasmic processes (Fig. 1a).

### Evaluation of proliferation ability of hPDLSCs

The proliferation rate and viability of the hPDLSCs was analyzed trough the MTT assay at passage 2. The examination of the acquired data showed a significant exponential cell growth during all times examined (Fig. 1b).

### Cytofluorimetric characterization of hPDLSCs

The expression of surface molecules CD13, CD29, CD44, CD73, CD90, CD105, CD146, and CD166, and of pluripotency-associated markers OCT3/4, SOX2, and SSEA4 were analyzed in xeno-free cultured hPDLSCs (Table 1). The hPDLSCs were negative for the subsequent markers CD14, CD34, CD45, CD117, CD133, CD144, CD271, and HLA-DR.

**Table 1:** Cytofluorimetric analysis of xeno-free Human periodontal ligament stem cells cultured at the second passage; the data are representative of five separate experiments

Stemness markers	Oct 3/4	97.2 ± 1.3 %
	Sox-2	98.4 ± 1.8 %
	SSEA-4	97.3 ± 1.6 %
Surface markers	CD 13	96.1 ± 1.3 %
	CD 29	97.1 ± 3.2 %
	CD 44	95.2 ± 2.1 %
	CD 73	96.2 ± 1.3 %
	CD 90	93.1 ± 3.1 %
	CD 105	97 ± 2.9 %
	CD 146	96 ± 2.8 %
	CD 166	95 ± 1.2 %
Hematopoietic markers	CD 14	Not detected
	CD 34	Not detected
	CD 45	Not detected
	CD 117	Not detected
	CD 133	Not detected
	CD 144	Not detected
	CD 271	Not detected
	HLA-DR	Not detected

### Mesengenic differentiation of hPDLSCs

Confluent living cells were induced to osteogenic differentiation, and after 3 weeks the cells were stained with Alizarin Red S to visualize the calcium deposits. The hPDLSCs showed a characteristic arrangement; the cells were detached from the bottom well and several areas of high levels of mineralization related to bone nodule were evident (Fig. 1c). The analysis of transcripts RUNX-2 and ALP confirmed the ability of the hPDLSCs towards osteogenic differentiation (Fig. 1d). To evaluate the adipogenic differentiation of hPDLSCs, the cellular monolayer was stained with Oil Red O and observed by light microscopy. Adipogenic-induced cells showed the well evident intracellular lipid droplets as single or grouped (Fig. 1e). These data were supported by upregulation, evaluated through real-time PC,R of the transcripts FABP4 and PPARγ, molecules that influence the biological pathway of adipogenesis (Fig. 1f). hPDLSC pellets cultured for 3 weeks under chondrogenic conditions showed strong Alcian blue staining (Fig. 1g), indicating abundant extracellular matrix proteoglycans and glycosaminoglycans. Moreover, hPDLSCs expressed high levels of chondrogenic differentiation-related genes, such as ACAN and COL2A1 (Fig. 1h).

### Clinical score and body weight

**Fig. 2:**
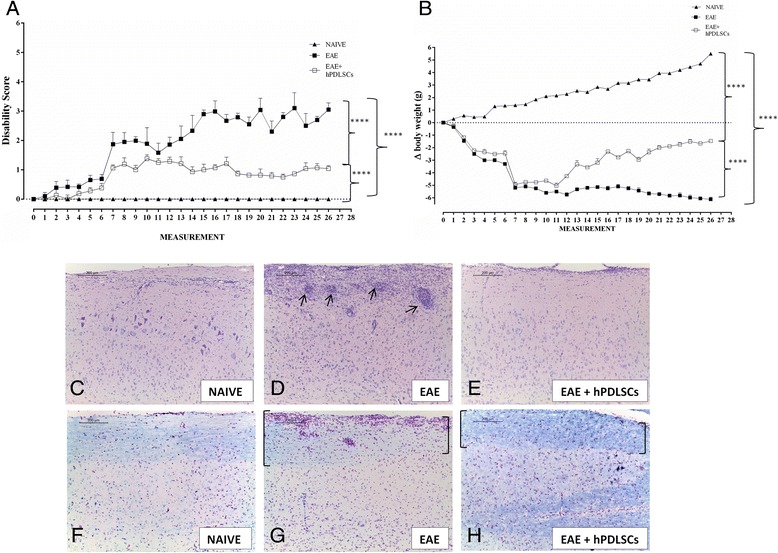
Clinical score, body weight, H&E and LFB. Mice were immunized with MOG_35–55_ and monitored every 2 days for clinical disease score of experimental autoimmune encephalomyelitis (*EAE*) and body weight gain/loss for 56 days. **a** Normal, naive mice did not display motor deficit. EAE mice displayed a grading of disease with a mean clinical score of 3.05, while mice treated with human periodontal ligament stem cells (*hPDLSCs*) revealed a lower grade of disability with a mean clinical score of 1.05. The measure of clinical disease score is expressed as mean ± SEM of all measurements of each experimental group. ****p < 0.0001. **b** The variation of body weight has been expressed compared to day of EAE induction (day 0) as mean ± SEM of all animals for each experimental group. Naive mice showed a normal increase in body weight. On the contrary, a significant body weight loss was observed in EAE mice, whereas a relevant body weight gain was found in hPDLSC-treated mice. ****p < 0.0001. hPDLSC treatment improved inflammatory cell infiltration caused by EAE. The severity of the damage was evaluated 56 days after EAE induction by H&E staining. **c** Naive mice did not show histological alterations in the spinal cord tissues. **d** H&E staining for EAE mice displayed a wide area of infiltrating cells (*arrows*) confirming the important role of the cellular components that undergoes degenerative conditions. **e** Conversely, treatment with hPDLSCs led to a complete resolution of inflammatory cells infiltration. **g** In addition, EAE untreated animals exhibited remarkably reduced myelin in the spinal cord (area in brackets), whereas naive mice did not show demyelination plaques (**c** and **f**). **h** Moreover, treatment with hPDLSC reduces demyelination and axonal loss in EAE mice with an intense LFB positive staining (see area in brackets)

Clinical disease score as well as body weight measurements were assessed as parameters of the disease. EAE is a well-characterized, validated and recognized model of MS in the mouse, which mimics the main features of the disease, including paralysis, weight loss, demyelination, inflammation in the CNS and the breakdown of the blood–brain barrier (BBB). Animals suffering from EAE tend to reduce their body weight as a result of anorexia and deficient fluid uptake. During the course of EAE, changes in body weight also reflect disease severity. Mice often lose a small amount of weight on the day following immunization. This appears to be due to the effects of the administered adjuvant and pertussis toxin. The weight loss continues with the progression of EAE severity, most likely due to paralysis and reduced food intake as well as high production of pro-inflammatory cytokines during the acute phase of inflammation. Our results showed that, as expected, mice belonging to the naive group showed a normal increase in body weight (change = +5.5 g). On the contrary, after EAE induction, a significant body weight loss was observed in EAE mice (–6.12 g). However, a slight body weight loss was found in the EAE + hPDLSC group (–1.50 g) compared with the naive group (Fig. 2b). Also, EAE animals showed a grading of disease with a mean clinical score of 3.05, while mice administered with hPDLSCs revealed a lower grade of disability with a mean clinical score of 1.05. Naive animals did not show motor deficits (Fig. 2a). These results suggest that treatment with hPDLSCs ameliorates clinical severity and improves functional recovery of EAE mice.

### hPDLSCs improve histopathology of EAE

The improvement in clinical score after hPDLSC administration could reflect decreased inflammatory cell infiltration into the CNS. Therefore, to identify the effect of hPDLSC treatment on the inflammatory cell influx, sections of spinal cords from EAE mice were stained with H&E and LFB in order to detect inflammatory cell infiltration and demyelination, respectively, at 56 days after EAE induction.

Naive mice did not show histological alterations in the spinal cord tissues (Fig. 2c). Notable damage was observed in the EAE mice, as demonstrated by the presence of a wide area of infiltrating inflammatory cells such as lymphocytes and polymorphonuclear cells in the white matter of spinal cord, confirming the important role of the cellular components that undergo degenerative conditions (Fig. 2d). Treatment with hPDLSCs instead led to a reduction in inflammatory cell infiltration, suggesting a protective effect on nervous tissues (Fig. 2e).

As EAE is a demyelinating disease, hPDLSC treatment was also evaluated for the protective action on myelin sheath integrity by LFB staining. Compared to naive mice (Fig. 2f), EAE untreated animals exhibited remarkably reduced myelin and axonal structures in the spinal cord (Fig. 2g). Consistently, corroborating this evidence, other authors have reported myelin and axonal loss along different white matter tracts in EAE mice, displaying a significant loss in LFB staining. In parallel, our evidence showed that treatment with hPDLSCs reduced demyelination and axonal loss in EAE mice with an intense LFB-positive staining (Fig. 2h).

Overall quantitative analysis performed on sections of spinal cord observed with an optical microscope at 20× showed that the percentage of area occupied by the myelin was about 0.017 % in EAE mice while in EAE + hPDLSC mice it was estimated at about 16,014 %. These images are representative of at least three experiments. The values shown are the mean of three different fields observed.

### hPDLSCs modulate production of Treg cells, CD4 and CD8α production

Regulatory T (Treg) cells are characterized by the expression of the transcription factor Forkhead box P3 (Foxp3). Treg cells play a pivotal role in keeping the inflammatory T cells, e.g., Th1 and Th17, in check and in maintaining self-tolerance and immune homeostasis.

**Fig. 3:**
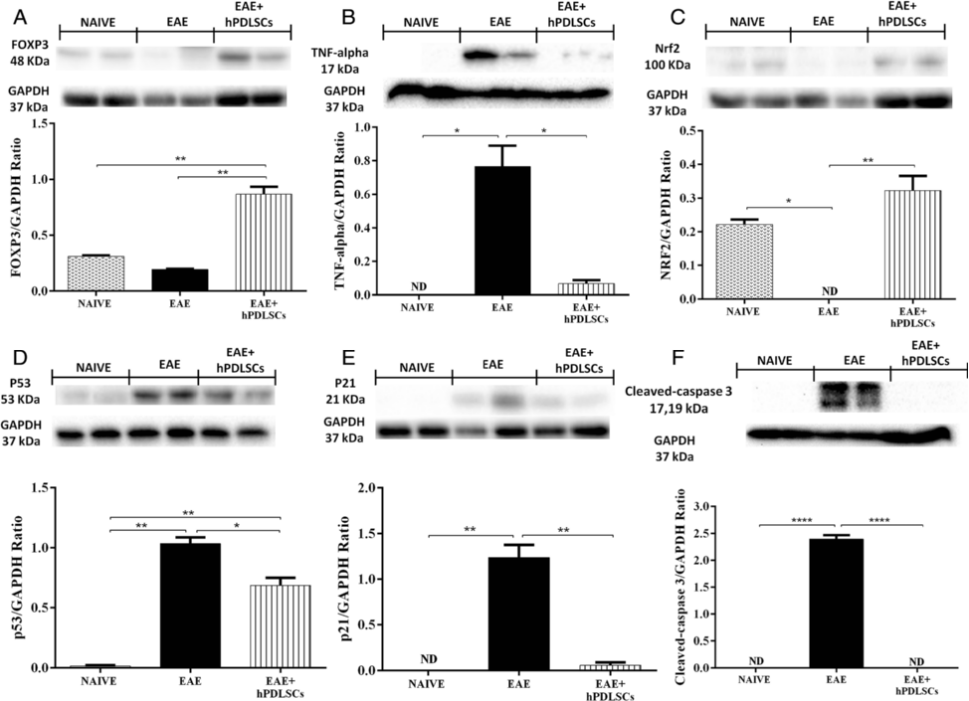
Western blot analysis for FOXP3, tumor necrosis factor (*TNF*)-α, Nrf2, cleaved caspase 3, p53 and p21. **a** Representative western blot showing no significant Foxp3 expression in spinal cord tissues from experimental autoimmune encephalomyelitis (*EAE*) mice as well as in the naive group. Foxp3 levels were appreciably increased in tissues from EAE mice administered with human periodontal ligament stem cells (*hPDLSCs*). **p < 0.0066 vs naive; **p < 0.0038 vs EAE. **b** Also evident is a notable increase in TNF-α release in samples collected 56 days after EAE induction, attenuated by administration of hPDLSCs. Naive mice did not show expression of TNF-α. *p < 0.0148 vs naive; *p < 0.0192 vs EAE. **c W**estern blot analysis for Nrf2 showed a basal level of Nrf2 expression in samples obtained from naive mice. EAE mice did not show expression of Nrf2, while treatment of mice with hPDLSCs significantly increased Nrf2 expression. *p < 0.0282 vs naive; **p < 0.0096 vs EAE. Western blot analysis for **d** p53 and **e** p21 showed a significant expression of p53 as well as p21 in samples collected 56 days after EAE induction when compared to the naive group. Conversely, levels of p53 and p21 were clearly reduced by administration of hPDLSCs. **p < 0.0018, **p < 0.0061 vs naive; *p < 0.0395 vs EAE; **p < 0.0052 vs naive; *p < 0.0060 vs EAE. **f** The activation of cleaved caspase 3 was evaluated. EAE caused a significant increase in cleaved caspase 3 expression. On the contrary, treatment with bioactive hPDLSCs prevented the EAE-induced caspase 3 expression. ****p < 0.0001 vs naive; ****p < 0.0001 vs EAE. GAPDH was used as the internal control. *ND* not detectable

**Fig. 4:**
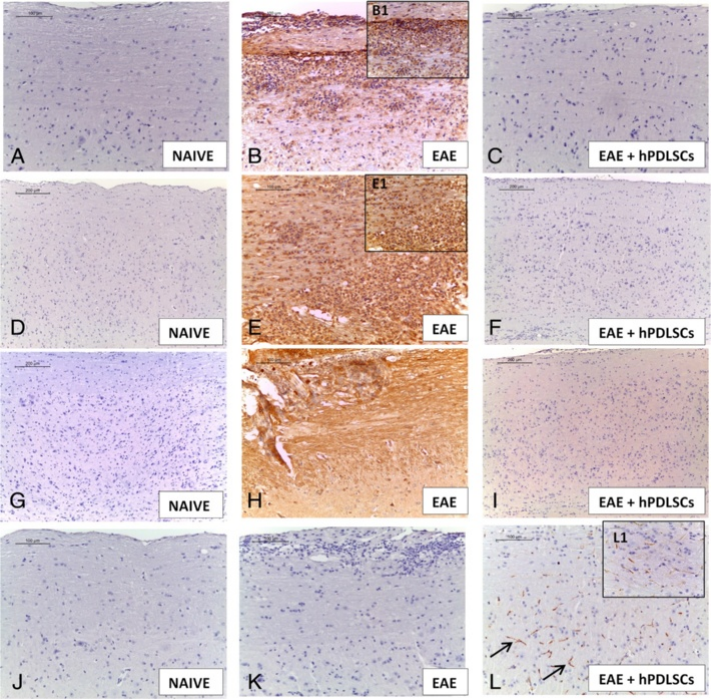
Immunohistochemical evaluation for CD4, CD8α, IL-1β and IL-10. CD4 and CD8α expression reveals higher tissue levels of these markers in experimental autoimmune encephalomyelitis (*EAE*)-affected mice (**b,** 20×, B1 magnification, 40×; **e**, 20×, E1 magnification, 40×) compared to the human periodontal ligament stem cell (*hPDLSC*)-treated group (**c**, **f**). Spinal cord specimens from naive mice did not stain for CD4 as well as for CD8 (**a**, **d**). Increased IL-1β tissue localization in EAE mice was reported (**e**, **h**). On the contrary, reduced expression of IL-1β was observed in mice that received hPDLSCs (**i**). **g** The immunohistochemical analysis showed that no positive staining for IL-1β was observed in the tissues obtained from naive mice. **k** EAE sections did not stain for IL-10 antibody. **l** Positive IL-10 expression levels were found in the EAE group treated with hPDLSCs (*arrows*; **l**, 20×, L1 magnification, 40×). **j** Naive mice showed negative staining for IL-10

To assess whether treatment with hPDLSCs was able to modulate the production of Treg cells, we evaluated expression of the transcription factor Foxp3 by western blot analysis. We observed no high expression of Foxp3 in animals subjected to EAE as well as in naive ones, while hPDLSC administration increased its expression (Fig. 3a). This observation has special relevance if we consider that CD4 T-cell expression is involved in cell-mediated immunity and in the pathogenesis of MS, with the destruction of the axonal myelin sheath in several areas of the CNS and spinal cord mediated mainly by self-reactive CD4 T cells. Immunohistochemical analysis carried out in spinal cord sections showed a positive staining for CD4 as well as for CD8α in EAE mice (Fig. 4b and e). Conversely, a negative staining for CD4 and CD8α was found in EAE mice administered with hPDLSCs (Fig. 4c and f) and in the naive group (Fig. 4a and d see densitometric analysis Fig. 6).

### hPDLSCs modulate the inflammatory pathway

Modulation of inflammatory mediators in mouse spinal cord, particularly with regard to two important cytokines altered in the MS patients’ profile, was investigated to understand and assess the effects of hPDLSC treatment on molecular mechanisms of inflammation. For this reason, the expression levels of IL-1β and TNF-α in spinal cord samples were quantified by immunohistochemical and western blot analysis.

Visibly, our results showed a negative staining for IL-1β in spinal cord sections from naive mice (Fig. 4g) as well as for EAE mice treated with hPDLSCs (Fig. 4i), while EAE mice that did not receive pharmacological treatment displayed positive staining for this pro-inflammatory cytokine (Fig. 4h see densitometric analysis Fig. 6).

By western blot analysis there was a considerable increase in TNF-α release over the course of EAE as evidenced in samples collected from EAE mice. On the contrary, reduced expression of TNF-α was observed in mice that received hPDLSC administration. TNF-α expression was not observed in naive animals (Fig. 3b).

A negative immunolocalization for IL-10 as an anti-inflammatory cytokine was found in spinal cord sections of EAE mice not treated with hPDLSCs (Fig. 4k); when hPDLSCs were given, on the contrary, IL-10 expression was kept at high levels, preserving tissues damaged by EAE (Fig. 4l). Naive mice showed negative staining for IL-10 (Fig. 4j see densitometric analysis Fig. 6).

### Effects of hPDLSCs on Nrf2 and GFAP expression

**Fig. 5:**
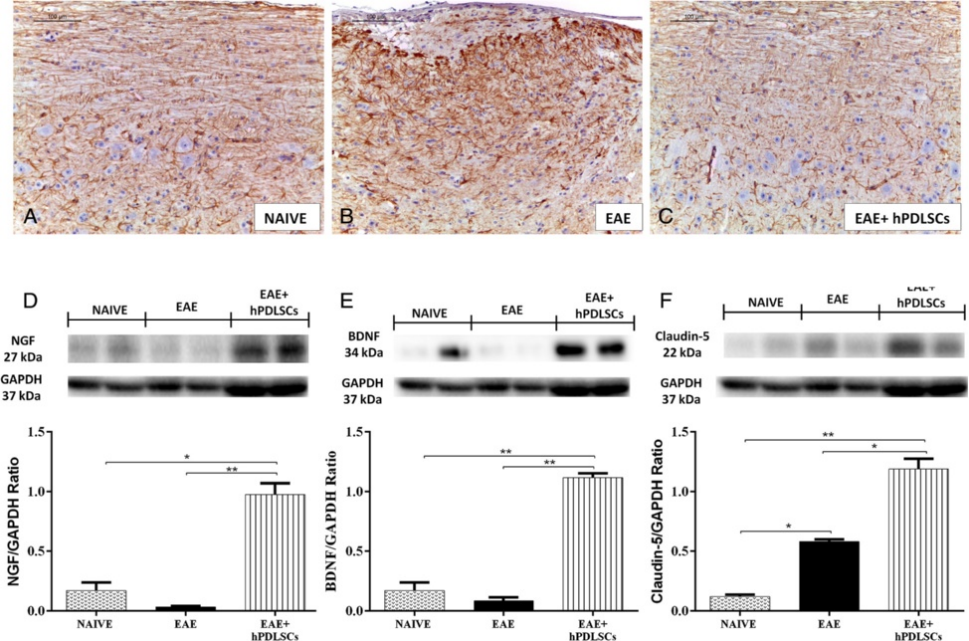
Immunohistochemical evaluation for GFAP, and western blot analysis for nerve growth factor (*NGF*), brain-derived neurotrophic factor (*BDNF*) and claudin-5. Immunohistochemical evaluation for GFAP tissue expression reveals an increased GFAP tissue localization in experimental autoimmune encephalomyelitis (*EAE*) mice (**b**) compared to the naive group (**a**). On the contrary, reduced expression of GFAP was observed in mice that received human periodontal ligament stem cells (*hPDLSCs*) (**c**). NGF and BDNF levels were appreciably increased in brain samples taken from mice administered with hPDLSCs, while mice subjected to EAE showed reduced levels of these neurotrophic factors (**d**, **e**). *p < 0.0103 vs naive; **p < 0.0065 vs EAE; **p < 0.0022 vs naive; **p < 0.0017 vs EAE. A basal level of claudin-5 production was found in brain samples collected from naive mice. Brain levels of claudin-5 increased in animals treated with hPDLSCs (**f**) more than in EAE mice. *p < 0.0251, **p < 0.0022 vs naive; *p < 0.0114 vs EAE. GAPDH was used as the internal control

**Fig. 6:**
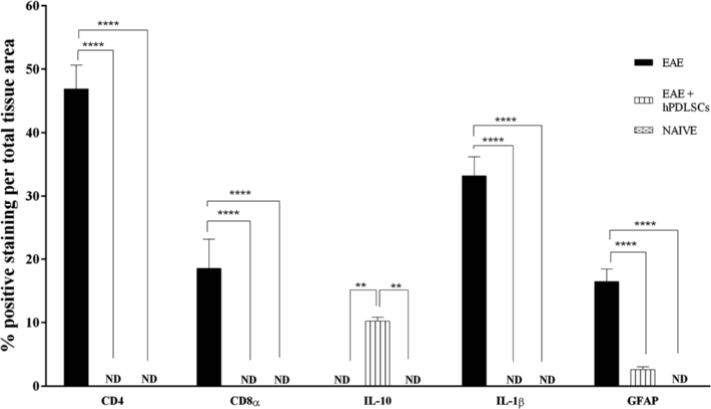
Densitometric analysis for CD4, CD8α, interleukin (*IL*)-1β, IL-10 and glial fibrillary acidic protein (*GFAP*). For immunohistochemical images, densitometric analysis was carried out to quantify and highlight significant differences among experimental groups. *p* value < 0.05 was considered significant. *ND* not detectable

Astrocytes are the major glial cell population within the CNS. After severe activation, astrocytes secrete various neurotoxic substances and express an enhanced level of GFAP, which is considered a marker protein for astrogliosis. With the aim to investigate whether hPDLSCs can modulate astrocytic activation during EAE, spinal cord sections were stained with an anti-GFAP antibody. Mice subjected to EAE exhibited positive immunolocalization for cytosolic GFAP (Fig. 5b), compared to the naive group (Fig. 5a). Treatment with hPDLSCs significantly reduced the degree of positive staining for GFAP (Fig. 5csee densitometric analysis Fig. 6).

These results were correlated with the expression of Nrf2, a transcription factor that binds to a short antioxidant response element, found in the promoters of a number of detoxification genes including those involved in redox homeostasis. The induction of Nrf2-mediated transcription, particularly in astrocytes, has been shown to protect against neurotoxicity from a variety of insults. Western blot analysis showed a basal level of Nrf2 expression in spinal cord samples obtained from naive mice. EAE mice did not show expression of Nrf2, but it was increased by treatment with hPDLSCs (Fig. 3c).

### hPDLSCs enhance neurotrophic factor release and restore BBB vascular endothelium after EAE induction

By western blot analysis we have detected NGF as well as BDNF expression in brain samples in order to demonstrate whether neuroprotective effects could be due to release of neurotrophic factors. The results clearly showed that EAE induction caused a decreased NGF expression, while, under the same damaging conditions, hPDLSC administration increased NGF levels (Fig. 5d). Likewise, our results indicate that hPDLSC administration following EAE enhanced BDNF levels, showing the same trend as the abovementioned neurotrophic factors (Fig. 5e). Naive mice also showed a basal level of NGF as well as BDNF expression (Fig. 5d and e).

During EAE the recruitment of inflammatory cells into the CNS parenchyma is accompanied by the breakdown of the BBB. Thus, in order to evaluate whether BBB breakdown is accompanied by the loss or alterations of tight junction (TJ)-associated molecules from the BBB, we investigated the claudin-5 expression by western blot analysis. A basal level of claudin-5 was found in brain samples collected from naive mice. Brain levels of claudins were increased in animals treated with hPDLSCs compared to EAE untreated mice (Fig. 5f).

### hPDLSC treatment inhibits EAE-induced apoptosis

Finally, we evaluated the degree of apoptosis associated with EAE, testing the role of hPDLSCs in attenuating cell death.

At 56 days after EAE, proteins in the mitochondrial p53 pathway and one of its target genes, p21, were detected by western blot analysis. p53 is able to induce apoptosis both by controlling the translation of pro-apoptotic p53-checked mediators and by nontranscriptional mechanisms, including upregulation of pro-apoptotic proteins and downregulation of anti-apoptotic mediators. EAE mice showed a significant expression of these markers, when compared to the naive group. Conversely, their expression was reduced by administration of hPDLSCs (Fig. 3d and e).

As known, sequential activation of caspases plays a central role in the execution phase of cell apoptosis, leading to programmed cell death by cleavage of cellular substrates. By western blot analysis, we evaluated the activation of cleaved caspase 3 in spinal cord tissues. Cleaved caspase 3 levels were appreciably increased in the spinal cord from mice subjected to EAE. On the contrary, treatment with hPDLSCs completely prevented EAE-induced cleaved caspase 3 expression. In naive animals cleaved caspase 3 expression was not observed (Fig. 3f).

## Discussion

This study aimed to investigate an alternative source of MSCs as a new treatment for autoimmune and neurodegenerative diseases such as MS.

Recently, we reported an innovative xeno-free culture system for the expansion of hPDLSCs demonstrating cell phenotype and genomic stability, essential elements to conform to cell-based therapy [14, 30].

The need to operate under strict Good Manufacturing Procedure and demonstrate feasibility and safety are the goals for cell-based therapy in clinical application [31, 32].

The multipotent capacity and the immunomodulatory properties both in vitro and in vivo of hPDLSCs make these cells a more accessible cell niche than bone marrow-derived stem cells for cell-based therapy of immune and inflammation-related diseases [33]. Moreover, hPDLSCs express the SDF-1a/CXCR4 complex, playing an essential role in homing of hematopoietic stem/progenitor cells [26, 34, 35] and driving the cells to the injury site. hPDLSCs do not express HLA-II DR [36], they display low immunogenicity, and can be used in autologous, allogenic and xenogenic transplantation. Thus, the possibility to derive pluripotent MSCs from an easily accessible, young and renewable cell source, such as human periodontal stem cells, could: i) dramatically reduce the quantity and quality issues involved in the use of adult tissue-derived MSCs; ii) prevent the need for constant donor recruitment (indeed the periodontal ligaments can be obtained from routine oral surgery); and iii) reduce potential risks from the use of multiple donors—in fact it could be desirable to bank the cells in an authorized structure.

Our data demonstrated that intravenous injection of hPDLSCs into mice immunized with MOG_35–55_ at the disease onset significantly improves clinical features such as disability score as well as body weight loss, which are well correlated with the severity of the pathology.

In agreement with earlier studies that MSCs limit demyelination in MS [37, 38], we found by performing histological evaluations of stained sections of the spinal cord that there were fewer inflammatory cell infiltrates and significantly less axonal loss in hPDLSC-treated mice.

Our evidence also demonstrated that hPDLSCs displayed notable immunomodulatory and anti-inflammatory capabilities. Regarding the molecular and cellular mechanism underlying EAE, firstly we looked at the state of activation of the immune system by evaluating the expression of CD4 and CD8α cells. Cells of both the innate and adaptive immune system initialize the neurodegenerative process via different effector molecules such as cytokines or reactive species [39]. As expected, we observed that both CD4 and CD8α detections were apparent in untreated EAE mice, while hPDLSCs show the capability to counteract the release of cytotoxic T cells.

These data have been further confirmed in hPDLSC-treated mice with a high and significant Foxp3 detection, as an indirect marker of Treg (also known as CD4+/FoxP3^+^) cell presence. Treg cells play a key role in controlling immune responses after a successfully defeated infection or to prevent autoimmune diseases [40, 41]. Therefore, treatment with MSCs can play a crucial role in protection against undesired T-cell activation and autoimmune disease by promoting Treg cell production. It seems that the beneficial effects of hDPLSCs on animals subjected to EAE could be due to their inherent properties to harness inflammatory cell infiltration, suppress inflammatory mediator production—as confirmed by a significant decrease in TNF-α expression as well as IL-1β over the course of treatment—and regulate immune tolerance by increasing the production of anti-inflammatory cytokines such as IL-10.

Moreover, inflammatory mediators have also been identified in the initiation of BBB breakdown. These mediators act via promoting the disruption of the TJ assembly, leukocyte recruitment and directly damaging the microvasculature. Among the various components of the BBB, the TJ claudin proteins are the most widely studied, and are critical for maintaining the BBB structural integrity and permeability [42]. The disruption of the cerebrovascular claudin-5 has been strongly correlated with the dynamic event of BBB breakdown [43]. In our study, we found that EAE induces changes in claudin-5 expression, and hPDLSCs modulate claudin-5 expression and control TJ permeability.

It is well known that astrocytes, the most abundant population of glial cells, are essential for brain homeostasis and maintenance and maturation of the BBB [44], but they are also capable of secreting inflammatory factors which aggravate the damage. Thus, we have demonstrated that EAE induced a notable increase in immunostaining for GFAP, considered as a marker of astrocytic reactivity, that in turn was significantly inhibited in the hPDLSC-treated group.

In addition, we evaluated the expression of Nrf2, known as the main transcription factor that regulates cellular defense mechanisms through antioxidant response elements in normal tissues [45]. The induction of Nrf2, particularly in astrocytes, has been shown to protect against neurotoxicity from a variety of injuries; in fact, several of the genes commonly regulated by Nrf2 have been implicated in protection from neurodegenerative conditions [46]. Our data showed that hPDLSC treatment leads to an upregulation of Nrf2 expression. In response, a protective action of Nrf2 on astrocytes occurs causing a downregulated GFAP expression.

Moreover, studies have revealed that apoptotic cell death of oligodendrocytes and/or neurons contributes to axonal injury and to other pathological events leading to neurological deficits linked to MS [47, 48]. In light of this, we studied the role of hPDLSCs on apoptosis. Specifically, we investigated a possible role of p53 and one of its target genes, p21, involved in the cell death process. p53 is an extremely important protein in determining cell fate decisions and its activation can result in the transcriptional induction of target genes that regulate apoptosis [49]. In line with these findings, EAE mice showed high levels of p53 and p21. On the contrary, hPDLSC treatment downregulated their expression.

Downstream pathways leading to intrinsic and extrinsic activation of the caspase proteases have been thoroughly described in EAE. Therefore, we evaluated the expression of cleaved caspase 3, and found that hPDLSCs inhibited EAE-induced apoptosis by decreasing the level of cleaved caspase 3. Therefore, we believe that hPDLSCs have the capability to interfere with EAE-induced neuronal cell death, attenuating or even preventing the activation of molecular pathways triggered by the injury.

Looking at all these results, we hypothesized that hPDLSC treatment may exhibit neuroprotection via activation of neurotrophic mediators which regulate crucial processes such as axonal growth and synaptic plasticity in CNS [50]. Interestingly, we found that hPDLSC administration increased the expression levels of NGF and BDNF factors.

## Conclusion

In light of the achieved results, we propose transplantation of hPDLSCs as a putative novel tool for treatment of autoimmune and neurodegenerative diseases such as MS. These cells could have considerable implication for future therapies for MS, and this study may represent the starting point for further investigations.

## Abbreviations

ACAN: Aggrecan
ALP: Alkaline phospatase
BBB: Blood–brain barrier
BDNF: Brain-derived neurotrophic factor
CNS: Central nervous system
COL2A1: Collagen type II
EAE: Experimental autoimmune encephalomyelitis
FABP4: Fatty acid binding protein 4
GFAP: Glial fibrillary acidic protein
H&E: Hematoxylin and eosin
hPDLSC: Human periodontal ligament stem cell
IL: Interleukin
LFB: Luxol Fast Blue
MOG: Myelin oligodendroglial glycoprotein peptide
MS: Multiple sclerosis
MSC: Mesenchymal stem cell
NGF: Nerve growth factor
PBS: Phosphate-buffered saline
PCR: Polymerase chain reaction
PPARγ: Peroxisome proliferator-activated receptor γ
RUNX-2: Runt-related transcription factor-2
TJ: Tight junction
TNF: Tumor necrosis factor
Treg: Regulatory T
